# Effects of a 15° Variation in Poll Flexion during Riding on the Respiratory Systems and Behaviour of High-Level Dressage and Show-Jumping Horses

**DOI:** 10.3390/ani13101714

**Published:** 2023-05-22

**Authors:** Paula Tilley, Joana Simões, José Paulo Sales Luis

**Affiliations:** 1 Centre for Interdisciplinary Research in Animal Health (CIISA), Faculty of Veterinary Medicine, University of Lisbon, Av. Universidade Técnica, 1300-477 Lisbon, Portugal; 2 Associate Laboratory for Animal and Veterinary Sciences (AL4AnimalS), 5000-801 Lisbon, Portugal; 3 Equine Clinical Academic Department, Faculty of Veterinary Medicine, Lusófona University, 1749-024 Lisbon, Portugal

**Keywords:** upper airways, over-ground endoscopy, conflict behaviour, dressage, show jumping

## Abstract

**Simple Summary:**

The impact of various degrees of poll hyperflexion on the welfare of ridden horses has previously been evaluated. The International Society for Equitation Science advised that lesser degrees of poll flexion should also be investigated. The aim of this study was to evaluate the effects of two degrees of poll flexion with a difference of only 15° on the respiratory system and behaviour of a horse during ridden exercise. Twenty high-level dressage and twenty show-jumping horses were ridden twice for 40 min 3 weeks apart, with the first ridden exercise at an 85° ground angle and the second at a 100° ground angle (the angle between the ground and the line from the forehead to the muzzle). Conflict behaviour was registered, as were manifestations of upper airway collapse, observed on an over-ground endoscopy. Arterial blood oxygen and lactate, pleural pressure, pharyngeal diameter, and heart and respiratory rates were evaluated. For both groups, at 100°, both conflict behaviours and upper airway abnormalities were seen more frequently, the intrathoracic pressure was higher, and the pharyngeal diameter was lower. At 85°, relaxation behaviours were more frequent. Compared to the first ridden exercise, the HR and RR were lower at the beginning of the second ridden exercise but higher at the end. The differences found here support the idea that an increase of just 15° in ridden poll flexion can have negative effects on the respiratory system and behaviour of a horse and therefore on its welfare.

**Abstract:**

From previous studies, the International Society for Equitation Science has advised that further research be conducted on the physiological/psychological effects of less-exacerbated poll flexion angles. We aimed to evaluate the effects of two riding poll flexion positions with a difference of only 15° on the respiratory systems and behaviour of horses through an evaluation of dynamic airway collapse via over-ground endoscopy, the pharyngeal diameter, pleural pressure, arterial oxygenation and lactate, HR/RR, and the occurrence of conflict behaviours. Twenty high-level dressage and twenty show-jumping horses underwent a 40 min ridden test at a ground angle of 85°; 3 weeks later, they underwent a ridden test at a 100° ground angle (the angle between the ground and the line from the forehead to the muzzle) and in a cross-over design. Using a mixed model for repeated measures, Wilcoxon/Friedman tests were carried out according to the experimental design and/or error normality. For both groups, at 100°, conflict behaviours and upper airway tract abnormalities were significantly more frequent, and the pleural pressure was higher, and the pharyngeal diameter was lower. At 85°, relaxation behaviours were significantly more frequent. Lactate was significantly higher at 100° only in the dressage horses. Compared to the first test at 85°, the HR/RR were significantly lower at the beginning of the second test (at 100°) but higher at the end. The significant differences identified in these dressage and show-jumping horses support the idea that an increase of just 15° in riding poll flexion can have negative effects on the respiratory system and behaviour of a horse and therefore on its welfare.

## 1. Introduction

Horses are elite athletes and have a much higher VO_2_ max (180 mL/kg/min) than humans (80 mL/kg/min), but they are obligate nasal breathers and are unable to switch to mouth breathing in order to decrease resistance. This causes a severe ventilatory limitation, thus constraining their athletic performance. Still, the horse’s respiratory system has some ways of decreasing resistance by dilating the external nares, abducting the larynx fully, and achieving some degree of bronchodilation. However, the friction and turbulence caused by the large increase in air speed during exercise compromises these adaptations. When we ride horses, we add to frictional resistance, often unknowingly. During exercise, horses generate very high air flows with resultant negative pressures inside their upper airways, risking their collapse. Therefore, they want to extend their heads and necks as this stiffens the trachea, preventing this collapse. In disciplines such as dressage and show jumping, we ask horses to bend the extra-thoracic airway at the level of the larynx and upper trachea, narrowing the lumen; in association with the increase in air flow speed during exercise, this leads to even greater negative intraluminal pressure in the airway according to Bernoulli’s principle, thus resulting in dynamic collapse and eventually lower air flows [1,2].

Public concern about the use of horses in sport has increased in recent years [3]. In addition, the lifespans of dressage and show-jumping horses are extremely important factors that are critical for success, as sport horses are usually at the top of their performance between 12 and 18 years of age. Therefore, any training technique that may affect a horse’s welfare and health in a negative way will influence the horse’s longevity and be contrary to the rider’s interests [4]. In wild horses, the angle between the dorsal nasal line and the ground is positively correlated with gait (and/or speed), but this is not the case in the ridden horse [5]. The hyperflexed position used in the ridden horse is not new [4], as there are cave paintings showing horses with crested necks, which can be exacerbated by the flexion of the neck. This can also be seen in pictures painted in some ancient Greek Chalcidian kraters from ca. 540 BC [4,6]. More recently, in the 1060s, it was reintroduced by the Schockemöhles in show jumping, and controversy about the use of this position began in the 1990s, when some dressage riders adapted it for training purposes [4]. The possible differences between the use of the hyperflexed position in either dressage or show jumping must be further investigated. “Rollkur” is described as the dorsal nasal line of the horse being behind the vertical by more than 10° when the horse is ridden [7]. This charm, which can be seen in horses with flexed necks, is encouraged by studies that report dressage judges preferring to focus their visual attention on the cranial half of the horse (the head, neck, and chest) [5]. Strangely, the prevalence of different head–neck positions in equine competitions was evaluated, and it was verified that a head behind the vertical was only penalised with lower marks in the lower competition levels but not in the higher levels [7,8].

We can easily control a horse’s head position by applying pressure to the bit, but this associates two different responses from the horse (deceleration and neck flexing) to just one signal (bit pressure). Therefore, it is against the principles of learning theory [5]. When we combine lowered head positions with submissive behaviour and impotence in escaping pressure, we introduce the concept of “learned helplessness” [9,10]. The horse, as a prey species, potentially suppresses the demonstration of obvious signs of pain in the presence of possible predators, making it difficult for us to assess it [11]. However, horses show conflict behaviour, which is the response presented when experiencing difficulty in coping with mental or physical discomfort and is usually manifested as some form of resistance to handling, training cues, and/or equipment [12,13].

The Fédération Equestre Internationale’s (FEI) description of the general principles of dressage and paradressage refers to the position of the horse’s head and neck at the collected gaits (walk, trot, and canter) being naturally dependent on their stage of training and, to some degree, on their conformation, stating that the nose should be slightly in front of the vertical, except for the moment at which the rider applies the aids, when the head may momentarily become more or less vertical.

The position statement of the International Society for Equitation Science (ISES) on the alterations of a horse’s head and neck posture in equitation, based on the substantial number of studies previously performed on the impact of hyperflexion on horse welfare, recommended that additional research should be performed on the physiological and psychological effects of lesser degrees of poll flexion and extension for which little is known [10]. In the present study, the authors evaluated the effects of two riding poll flexion positions with only a 15° difference on the respiratory systems and behaviour of high-level dressage and show-jumping horses through an evaluation of dynamic airway collapse, pharyngeal diameter, pleural pressure, arterial oxygenation and lactate level, and the occurrence of conflict behaviour. The presence of significant differences between the dressage and show-jumping horses regarding the evaluated outcomes was also explored.

## 2. Materials and Methods

The study was approved by the Ethics Committee of the Faculty of Veterinary Medicine (University of Lisbon) and the legal owners of all the show-jumping team horses. Additionally, the Portuguese School of Equestrian Arts (EPAE—Escola Portuguesa de Arte Equestre) issued institutional consent for each horse that entered the study, which followed a cross-over design.

The sample size was estimated to identify an effect size of 5% between the two riding tests with two different poll flexion angles in dressage and in show-jumping horses, using an alpha of 0.05 and a statistical power of 80%. The drop-out rate was assumed to be near 0% as no horses were privately owned. The confirmation of the sample size estimation was carried out through a literature search directed at similar studies published previously with similar outcomes (outcome for behaviour—sample size of 7 [12], sample size of 15 [14]; outcome for pharyngeal diameter—sample size of 15 [15,16]; outcomes for heart rate and arterial blood gases—sample sizes of 8 [17,18] and 16 [19]; outcome for pleural pressure—sample size of 7 [18]; outcome for dynamic upper airway dysfunctions—sample size of 14 [20]).

**Figure 1:**
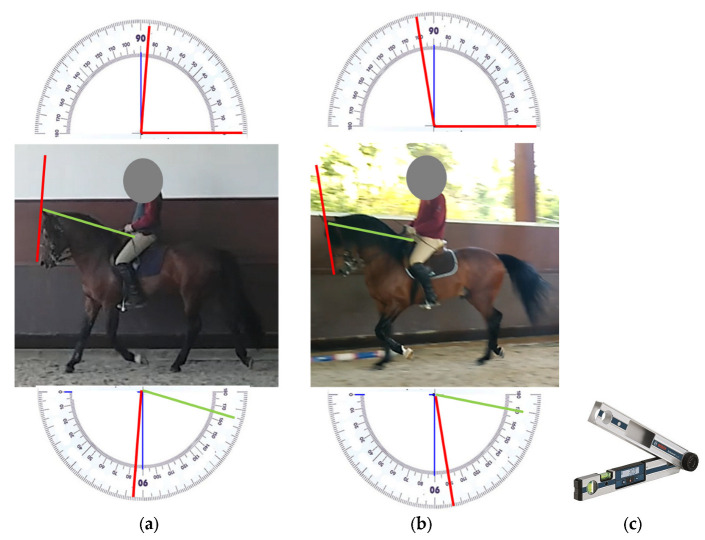
Ground angle: (**a**) 85° ground angle and 80° withers angle (green); (**b**) 100° ground angle and 70° withers angle (green); (**c**) Bosch DAF 220 K digital protractor.

Twenty *haute école* (high-level dressage) horses from EPAE, all Pure Blood Lusitano (PSL) Alter Real horses, and twenty average-level show-jumping horses (six PSLs, seven cross-PSLs, three Holsteins, two KWPNs, and two Selle Français horses) with no history of respiratory or cardiac pathology and with no concomitant lameness were included. Each horse underwent a 40 min standardized exercise test twice (the first test and the second test) while being ridden in an indoor arena by an experienced professional rider on 2 distinct days 3 weeks apart. These tests consisted of a 10 min walk, a 15 min trot, and a 15 min canter. In the 1st test, the horses were ridden with poll flexion corresponding to a ground angle of 85° and a withers angle of 80–82°. In the 2nd test, the horses were ridden with poll flexion corresponding to a ground angle of 100° and a withers angle of 67–70° (Figure 1a,b). The ground angle is the angle between the line from the forehead to the muzzle and the ground [21]. The withers angle is the angle between the line from the forehead to the muzzle and the line connecting the neck (starting at the atlanto-occipital joint) and the withers [21]. As previously recommended, to reduce the psychophysiological influences associated with the tests’ protocols, the horses were exposed to the indoor arena environment on one occasion prior to data collection [22]. This exposure also included the personnel involved. Furthermore, in order to limit the effects of diurnal variations on pulmonary mechanics, investigations always occurred at approximately the same time of day [23].

All riders were shown the intended flexion angles with the help of a Bosch DAF 220 K digital protractor (Figure 1c) before beginning the exercise tests. Two veterinarians then observed the horses thoroughly during the riding tests and ensured that the intended angles were maintained for the full length of each test. For each horse (show-jumping and dressage), both exercise tests were videotaped, and confirmation that the desired poll flexion angles (ground angle and withers angle) were maintained throughout the full tests was obtained by taking measurements on still film every 5 min for a total of seven times per film.

**Figure 2:**
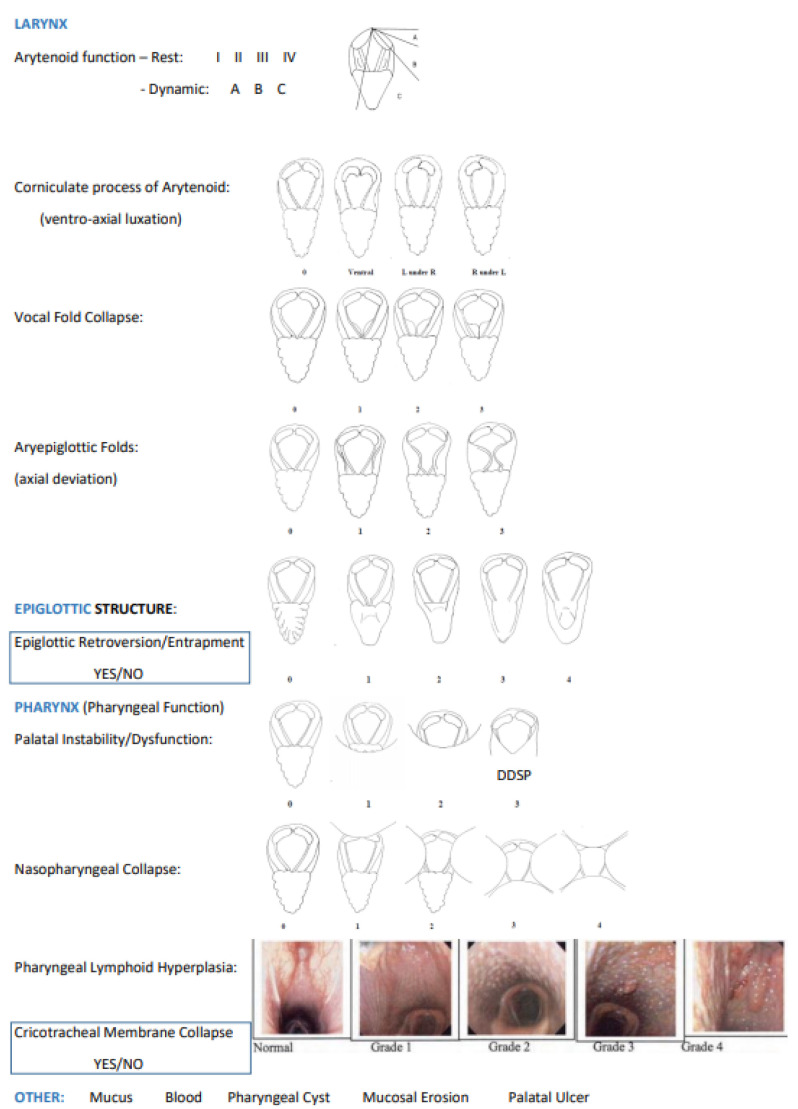
Over-ground endoscopy video classification, based on Holcombe (2005) and McGivney et al. (2017).

The occurrence of conflict behaviour was registered by two independent clinicians who reviewed the first 35 min of the exercise test film. The scoring system used to evaluate the horses’ behaviour (Table 1) was based on Smiet et al. (2014) [12] and von Borstel et al. (2009) [14]. Behaviours were classified as 0 if absent, 1 if occasional, 2 if moderately frequent, or for longer periods, 3 if considerably frequent or prolonged, and 4 if constant or continuous. For the last 5 min of the canter, the horses were subjected to an over-ground endoscopy (DRS PREMIUM Dynamic Endoscopy System, OPTOMED, 6 Avenue des Andes, Bâtiment 6, 91940 Les Ulis, France) in order to investigate and classify dynamic upper airway dysfunctions (Figure 2, based on McGivney et al., 2017 [24], and Holcombe, 2005 [25]) via the endoscopy videos. All videos were viewed twice by two independent experienced clinicians who were blinded to the horse identification and to the exercise test. The over-ground endoscope was only placed in the horses for the last 5 min so that its presence interfered as little as possible with the horses’ ridden work and poll flexion. For this purpose, the horses were briefly stopped at 35 min, and a team was ready to quickly place the over-ground endoscope.

The heart rate (HR) and respiratory rate (RR) were evaluated immediately before and immediately after both exercise tests, while the horses were in the arena.

Immediately after each exercise test, arterial blood was also collected in heparinized syringes from the transverse facial artery to measure the pH, BE, PO_2_, sO_2_, PCO_2_, TCO_2_, HCO_3_, and lactate using an i-STAT (i-STAT Handheld System, Abbott Point of Care Inc., 400 College Road East, Princeton, NJ 08540, USA) portable device with a CG4+ test cartridge, and the PaO_2_ and PaCO_2_ values were corrected for core body temperature [18]. The i-STAT analyser allows for the direct measurement of the pH, PaO_2_, PaCO_2_, and lactate, while the remaining parameters were calculated by the software. Baseline values for these parameters were not evaluated due to financial constraints.

As one veterinarian was waiting for the i-STAT results, the pleural pressure (ΔPpl) was immediately measured by another veterinarian using a Ventiplot (Ventiplot, Klinik für Pferde, Veterinärmedizinische Universität Wien, Vetmeduni Vienna, Veterinärplatz 1, 1210 Vienna, Austria) with an oesophageal balloon catheter technique. The oesophageal balloon catheter was placed in the distal third of the oesophagus (intrathoracic oesophagus). It was then connected to the pressure transducer, and 5 mL of air was used to inflate the balloon. The ΔPpl was recorded for each horse as the difference between the pressure during inspiration and expiration, and the average of 3 measurements was used. Artefacts caused by swallowing were excluded from the ΔPpl calculations.

After these procedures were concluded, lateral pharyngeal radiographs [21] were obtained using an Optimus X-ray generator (Optimus, Philips Healthcare, P.O.Box 10.000, 5680 DA Best, The Netherlands) and a Vertical Bucky (Vertical Bucky, Philips Bucky Diagnost, Philips Healthcare, P.O.Box 10.000, 5680 DA Best, The Netherlands ) with a focus distance of 1.20 m and an exposure factor of 109 kV/2 mAs in order to evaluate the pharyngeal diameter (as the pharyngoepiglottic diameter: the shortest distance between the dorsal aspect of the epiglottis and the roof of the pharynx) for both head and neck positions. For this measurement, the magnification correction factor for midsagittal structures was determined [26].

SPSS was used for statistical analysis. The data normality was studied; once data normality was achieved, a mixed model for repeated measures was adjusted. In this model, when sphericity was not attained by the Mauchy test, the Greenhouse–Geiser test was used. Otherwise, a standard ANOVA was used. For the variables that did not show error normality, the Wilcoxon test was used to compare distributions except for RR and HR, for which the Friedman test was used as there were more than 2 groups. Multiple mean comparisons were carried out using the Wilcoxon test when the Friedman results showed differences. A general discriminant analysis was then carried out to evaluate if it was possible to discriminate between the behaviours demonstrated with the first and the second head positions and between the two equestrian disciplines and to determine which variables were more important in these discriminations.

## 3. Results

### 3.1. Parameters Evaluated during the Ridden Exercises of the Two Tests

#### 3.1.1. Confirmation of Poll Flexion Angles (Ground Angle and Withers Angle)

**Figure 3:**
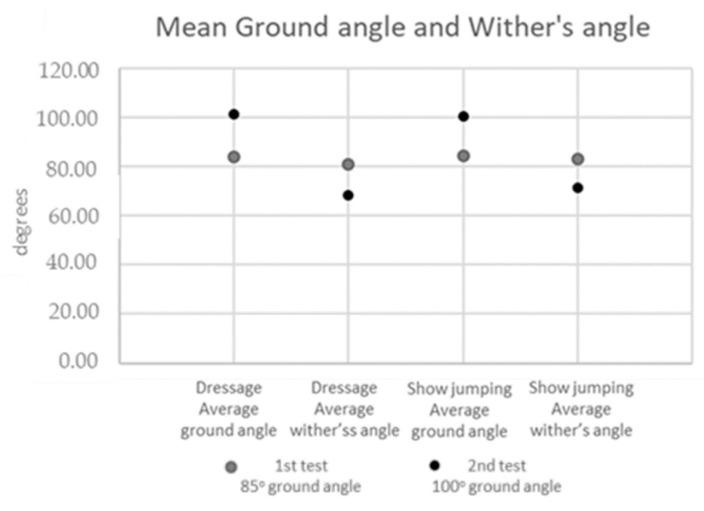
Mean ground angles and withers angles of dressage and show-jumping horses in the first and second tests.

In the first test, with the desired poll flexion at an 85° ground angle and a withers angle of 80–82°, the average of the measured ground angles was 84.15° ± 2.0 in the dressage horses and 84.50° ± 1.96 in the show-jumping horses. This corresponded to an average measured withers angle of 80.95° ± 1.83 in the dressage horses and 82.90° ± 2.27 in the show-jumping horses (Figure 3).

In the second test, with the desired poll flexion at a 100° ground angle and a 67–70° withers angle, the average of the measured ground angles was 101.40° ± 2.01 in the dressage horses and 100.50° ± 2.52 in the show-jumping horses. This corresponded to an average of the measured withers angles of 68.35° ± 2.98 in the dressage horses and 71.45° ± 2.24 in the show-jumping horses (Figure 3).

#### 3.1.2. Conflict Behaviour

Occurrences of various conflict behaviours were significantly higher with the 100° ground angle poll flexion position when compared to the 85° position in both equestrian disciplines.

**Figure 4:**
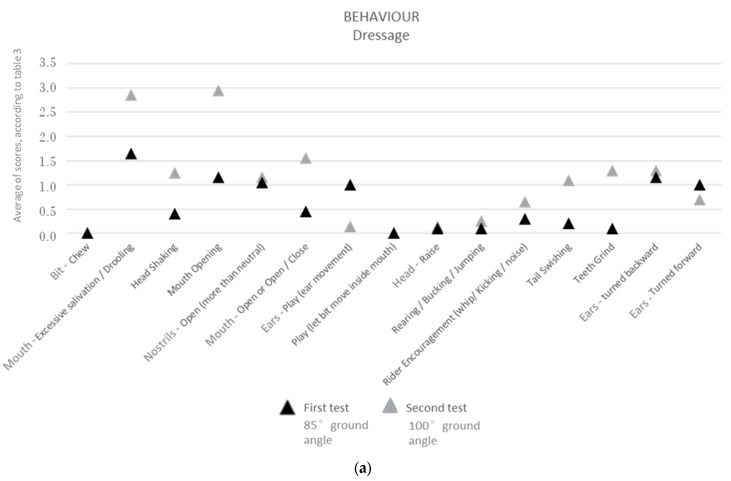

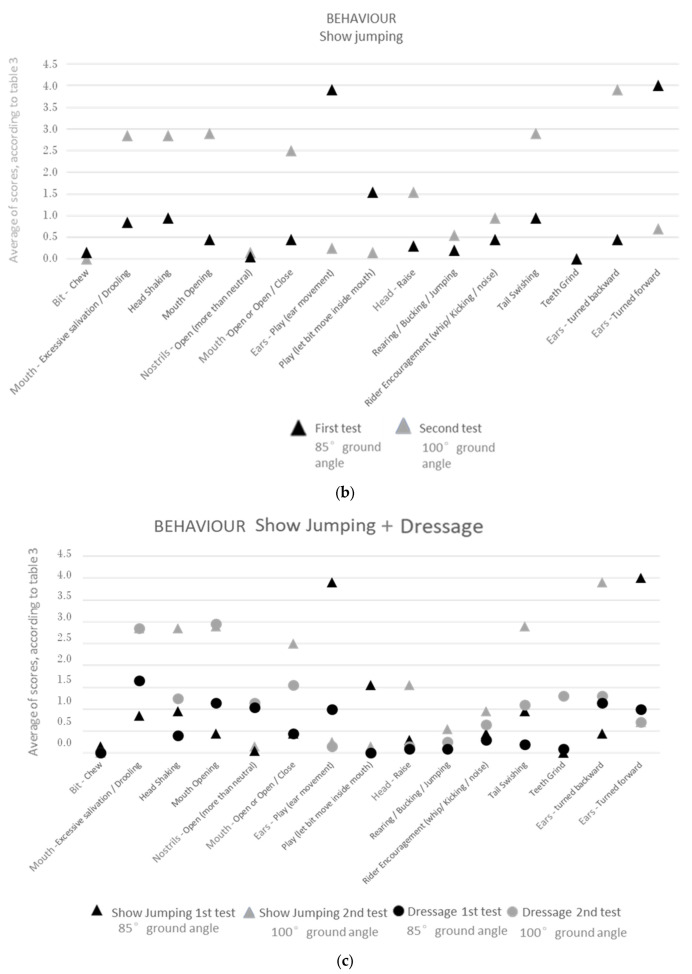
Behaviours shown by horses during the exercise tests in the dressage horses (**a**), in the show-jumping horses (**b**), and in show-jumping + dressage horses (**c**). First exam with the head flexed at a ground angle of 85°. Second exam with the head flexed at a ground angle of 100°.

For the dressage horses, the conflict behaviours that were significantly more common at 100° of poll flexion were tail swishing, head shaking, mouth opening, teeth grinding, mouth opening/closing (jaw movement) and excessive salivation/drooling. Rider encouragement (using the whip/kicking/producing noises) was also significantly more common with the 100° ground angle position. On the contrary, the relaxation behaviours occurred significantly more with 85° of poll flexion, and these behaviours were ear play (ear movement) and having the ears turned forward (Figure 4, Table 2).

For the show-jumping horses, the conflict behaviours that were significantly more common at 100° of poll flexion were tail swishing, head shaking, mouth opening, turning the ears backward, raising the head, mouth opening/closing (jaw movement), excessive salivation/drooling, and rearing/bucking/jumping. Still, the use of rider encouragement (whip/kicking/noise) was not significantly different between the two head positions. On the contrary, the relaxation behaviours occurred significantly more with 85° of poll flexion, and these were ear play (ear movement), having ears turned forward, and playing with the bit (letting the bit move inside the mouth), (Figure 4, Table 2).

When comparing the two equestrian disciplines, the conflict behaviours tail swishing, head shaking, mouth opening, mouth opening/closing (jaw movement) and excessive salivation/drooling occurred significantly more with the 100°-ground-angle poll flexion in both dressage and show-jumping horses. Similarly, the relaxation behaviours ear play (ear movement) and having the ears turned forward occurred significantly more with the 85°-ground-angle poll flexion position in both disciplines.

Rider encouragement (whip/kicking/noise) and the teeth-grinding conflict behaviour only occurred significantly more with the 100°-ground-angle poll flexion position in the dressage horses.

On the other hand, the conflict behaviours turning the ears backward, raising the head, and rearing/bucking/jumping only occurred significantly more with the 100°-ground-angle poll flexion in the show-jumping horses. Regarding the relaxation behaviours, such as playing with the bit (letting the bit move inside the mouth), also only occurred significantly more with the 85°-ground-angle poll flexion in the show-jumping horses.

Furthermore, when we look only at the conflict behaviours that were significantly more common at the 100°-ground-angle poll flexion than at 85° in both the dressage and show-jumping horses, we can see that tail swishing, head shaking, and having the ears turned backwards occurred significantly more often with the 100° ground angle in the show-jumping horses (*p* < 0.05) than in the dressage horses.

Likewise, considering only the relaxation behaviours that were significantly more common at the 85°-ground-angle poll flexion that at 100° in both equestrian disciplines, we can see that ear play (ear movement), having the ears turned forward, and playing with the bit occurred significantly more often with the 85° ground angle in the show-jumping horses (*p* < 0.05), than in the dressage horses.

**Figure 5:**
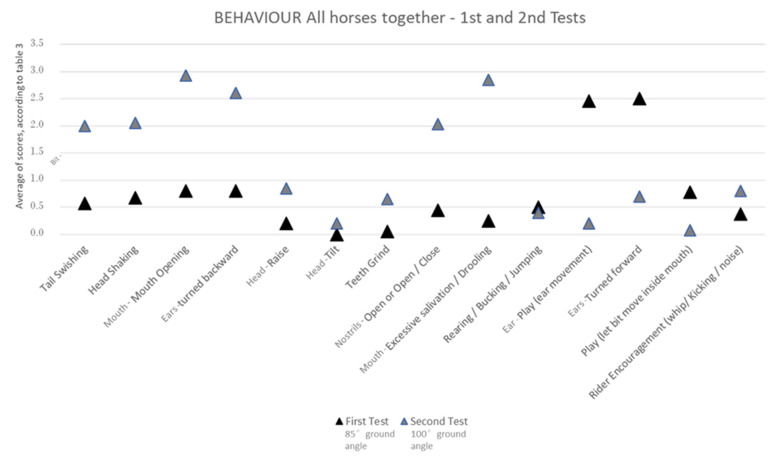
Behaviours shown by all horses together during the first exam, with the head flexed at a ground angle of 85°, and the second exam, with the head flexed at a ground angle of 100°. Significantly different behaviours are shown.

When looking for the significantly different conflict and relaxation behaviours between the 85°- and the 100°-ground-angle poll flexion positions, the observation of these differences becomes clearer when we evaluate all the horses at the same time (Figure 5, Table 2) than when we evaluate them in two groups (dressage and show-jumping groups) (Figure 4c, Table 2) or group by group (Figure 4a,b, Table 2).

**Figure 6:**
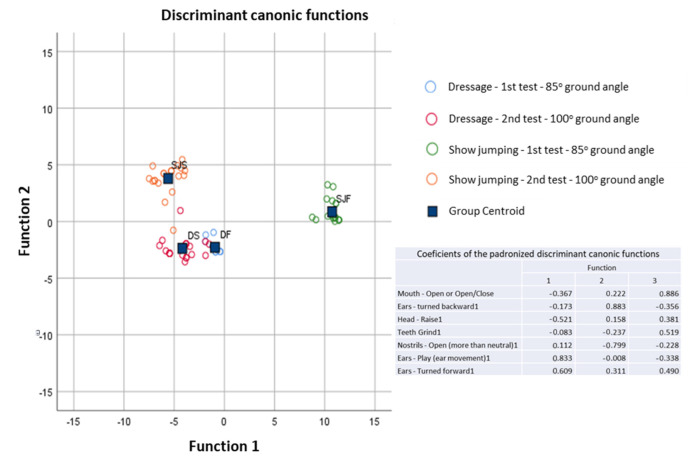
Behaviour-discriminant canonical functions for dressage and show-jumping horses in the two exercise tests (first test—85° ground angle; second test—100° ground angle).

The discriminant analysis (Figure 6) showed that it was possible to differentiate between the four groups (first and second head positions and show-jumping and dressage horses) solely via some of the behaviour variables observed during the exercise tests. These variables were the conflict behaviours mouth opening, having the ears turned backwards, head raising, teeth grinding, and nostril opening (more than neutral), as well as the relaxation behaviours ear play (ear movement) and having the ears turned forward. It was possible to correctly classify 95.0% of the originally grouped cases and 93.8% of the cases grouped by cross-validation. In the cross-validation, each case was classified by the derivative functions of all cases different from that case.

#### 3.1.3. Over-Ground Endoscopy—Upper Airway Tract Dynamic Dysfunctions

In the over-ground endoscopy, multiple upper airway tract dynamic dysfunctions were more commonly associated with the 100°-ground-angle poll flexion position in both the dressage and show-jumping horses.

**Figure 7:**
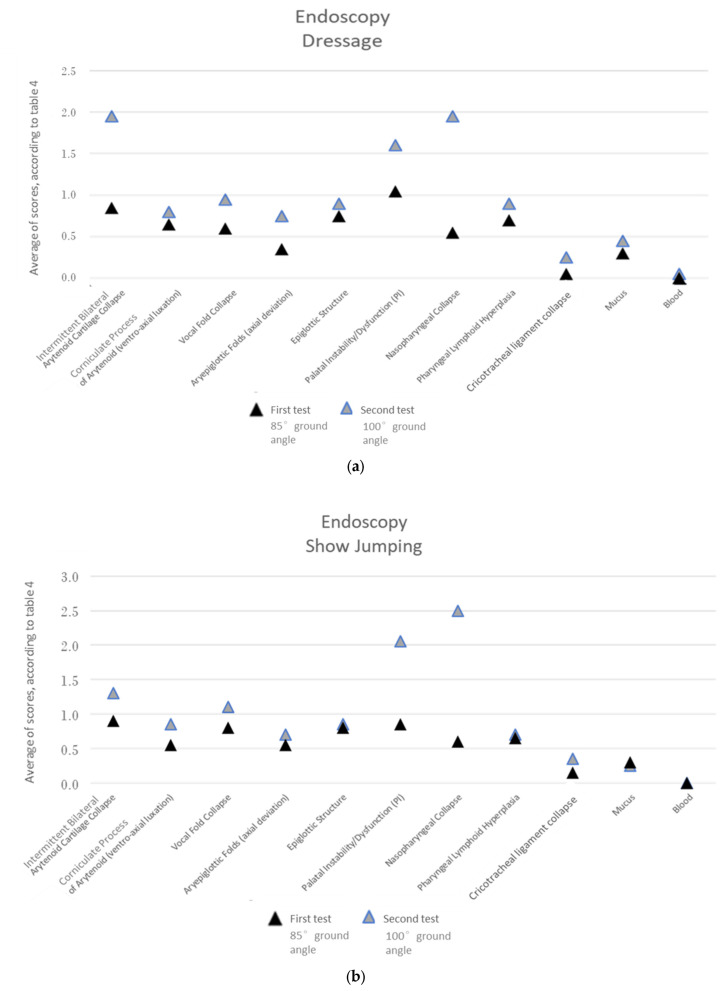

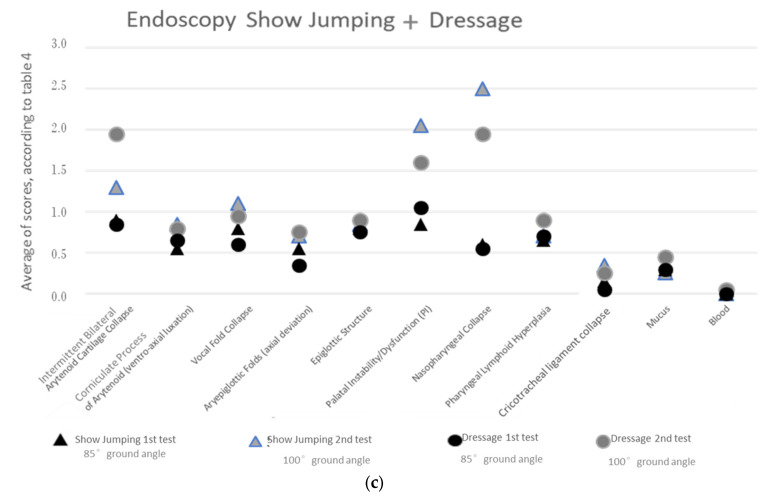
Upper airway tract dysfunctions detected via over-ground endoscopy during exercise in the dressage horses (**a**), in the show-jumping horses (**b**), and in show-jumping + dressage horses (**c**). First test with poll flexion at a ground angle of 85°. Second test with poll flexion at a ground angle of 100°.

In the dressage horses, this position showed a significantly higher occurrence of an aryepiglottic fold axial deviation, palatal instability/dysfunction, pharyngeal lymphoid hyperplasia, and the collapse of various structures (nasopharyngeal, vocal fold, intermittent bilateral arytenoid cartilage, and cricotracheal ligament collapse) (Figure 7, Table 3).

In the show-jumping horses, this position showed a significantly higher occurrence of an aryepiglottic fold axial deviation, palatal instability/dysfunction, ventroaxial luxation of the arytenoid corniculate process, and the collapse of various structures (nasopharyngeal, vocal fold, intermittent bilateral arytenoid cartilage, and cricotracheal ligament collapse) (Figure 7, Table 3).

When comparing the two equestrian disciplines, ventroaxial luxation of the arytenoid corniculate process only had a significantly higher occurrence with the 100°-ground-angle poll flexion position in the show-jumping horses and pharyngeal lymphoid hyperplasia in the dressage horses.

**Figure 8:**
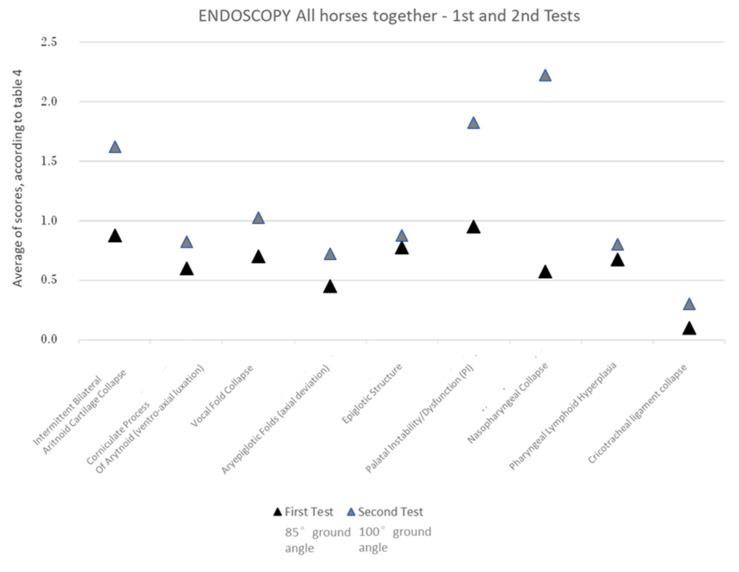
Upper airway tract dysfunctions detected via over-ground endoscopy during exercise in all horses together during the first exam, with the head flexed at a ground angle of 85°, and the second exam, with the head flexed at a ground angle of 100°. Significantly different dysfunctions are shown.

When looking for the significantly different upper airway dynamic dysfunctions between the 85°- and the 100°-ground-angle poll flexion positions, the observation of these differences becomes clearer when we evaluate all the horses at the same time (Figure 8, Table 3) than when we evaluate them in two groups (dressage and show-jumping groups) (Figure 7c, Table 3) or group by group (Figure 7a,b, Table 3).

The occurrence of cases of multiple upper airway dynamic dysfunctions in the same horse was registered for both equestrian disciplines.

### 3.2. Parameters Evaluated after the Ridden Exercise

#### 3.2.1. Arterial Blood

In the dressage horses, the blood lactate level was significantly higher with the 100°-ground-angle position (0.438 ± 0.28 mmol/L at 85° and 0.574 ± 0.44 mmol/L at 100°; *p* < 0.001), but this was not the case in the show-jumping horses, where it was not significantly different between the two poll flexion ground angles (0.44 ± 0.21 mmol/L at 85° and 0.51 ± 0.20 mmol/L at 100°; *p* = 0.638).

When considering the blood lactate values of all horses together, the level was again significantly higher when the 100°-ground-angle position was used (0.4471 ± 0.17 mmol/L at 85° and 0.574 ± 0.44 mmol/L at 100°; *p* = 0.049).

None of the other arterial blood parameters showed significant differences between the tests for any of the equestrian disciplines studied, nor for all horses together.

#### 3.2.2. Pleural Pressure (ΔPpl)

**Figure 9:**
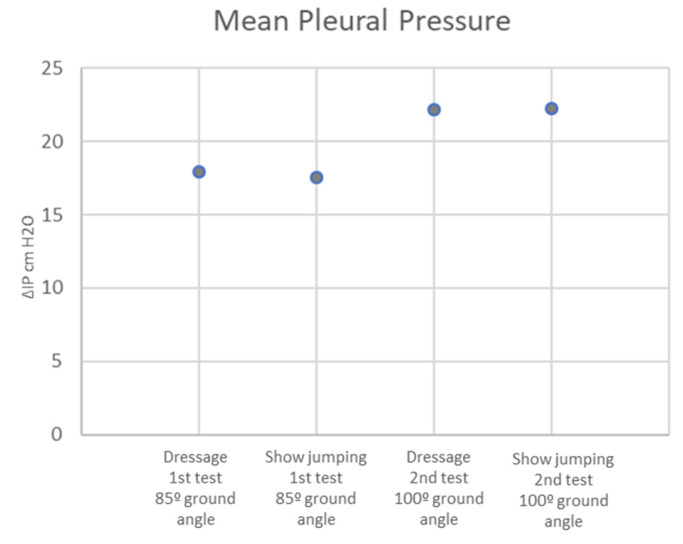
Mean pleural pressure for dressage and show-jumping horses after the first and second tests.

In the dressage horses, the difference in pleural pressure (ΔPpl) was significantly higher when the 100°-ground-angle position was used, as the ΔPpl at an 85° ground angle was 17.95 ± 1.14 cm H_2_O, and at 100°, it was 22.15 ± 1.04 cm H_2_O (*p* < 0.001) (Figure 9).

In the show jumping horses, pleural pressure (ΔPpl) difference was significantly higher in the 100°-ground-angle position, as ΔPpl at an 85° ground angle was 17.55 ± 2.01 cm H_2_O, and at 100°, it was 22.3 ± 2.61 cm H_2_O (*p* < 0.001) (Figure 9).

#### 3.2.3. Pharyngeal Diameter

**Figure 10:**
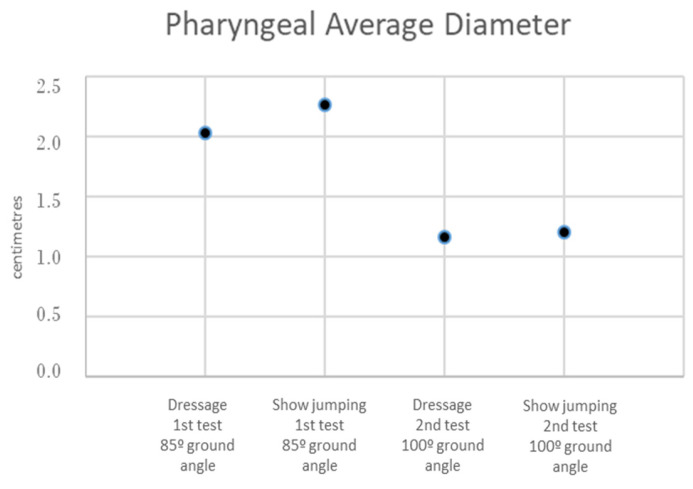
Mean pharyngeal diameter in the two tests for dressage and show-jumping horses.

**Figure 11:**
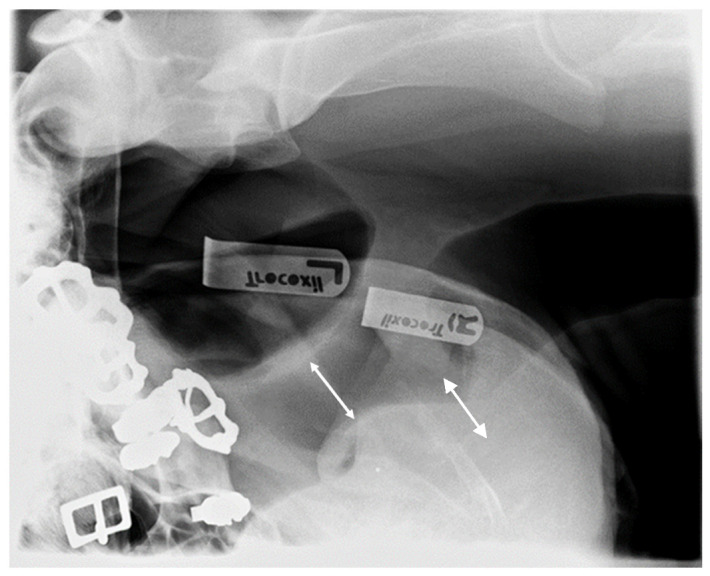
Measurement of pharyngeal diameter as the pharyngoepiglottic distance (white arrow) on the X-rays.

In the dressage horses, the pharyngeal diameter (2.028 ± 0.65 cm at 85° and 1.162 ± 0.50 cm at 100°) was significantly lower in the 100°-ground-angle position (*p* < 0.001) (Figure 10 and Figure 11).

In the show-jumping horses, the pharyngeal diameter (2.26 ± 0.79 cm at 85° and 1.21 ± 0.51 cm at 100°) was also significantly lower in the 100°-ground-angle position (*p* < 0.001) (Figure 10 and Figure 11).

### 3.3. Parameters Evaluated before and after the Ridden Exercise

#### Heart and Respiratory Rates (HR and RR)

Regarding the HR and RR, both were significantly lower at the beginning of the second exercise test when compared to the beginning of the first, although this difference was more noticeable in the show-jumping horses (Table 4).

Nevertheless, they were both significantly higher at the end of the second exercise test when compared to end of the first exercise test (Table 4).

Furthermore, significant differences were found for the HR but not for the RR among the two equestrian disciplines studied.

## 4. Discussion

Even though a lesser degree of flexion of just behind the vertical (as opposed to Rollkur) was used in the present study, as advised by ISES [10], the **occurrence of conflict behaviours** was more common when the 100°-ground-angle position was used for both equestrian disciplines studied. The conflict behaviours tail swishing, head shaking, mouth opening, mouth opening/closing (jaw movement), and excessive salivation/drooling occurred significantly more with the 100° ground angle00°-ground-angle poll flexion in both dressage and show-jumping horses.

Regarding relaxation behaviours, they occurred more commonly when the 85°-ground-angle poll flexion position was used for both disciplines. The relaxation behaviours ear play (ear movement) and having the ears turned forward occurred significantly more with the 85°-ground-angle poll flexion in both equestrian disciplines.

The following authors have reported the occurrence of conflict behaviours when some greater extent of poll flexion is used. Some found that with a coercively obtained Rollkur position, horses moved slower and showed behavioural signs of discomfort more often [14]. Others reported that the horses competing in dressage were ridden more often in both a low-head-and-neck position and with the nose behind the vertical, in contrast to the show-jumping horses [13].

In the present study, a few differences in the prevalence of behaviours were also found between the dressage and the show-jumping horses.

The conflict behaviour that occurred significantly more when the 100°-ground-angle poll flexion position was used only in dressage horses was teeth grinding; however, in this discipline, rider encouragement (whip/kicking/noise) was also significantly more present in this poll flexion position. In these dressage horses, the most frequent conflict behaviours with the 100°-ground-angle poll flexion were mouth opening and excessive salivation/drooling. This is slightly different from the results obtained in other studies, which reported that tail swishing was the most frequent conflict behaviour in a group of dressage horses [13].

The conflict behaviours that occurred significantly more with the 100°-ground-angle poll flexion position only in show-jumping horses were turning the ears backward, raising the head, and rearing/bucking/jumping. The the most frequent conflict behaviours in show-jumping horses with the 100°-ground-angle poll flexion position were having the ears turned backwards, tail swishing, head shaking, and mouth opening and excessive salivation/drooling. This is mostly in agreement with previous studies, which reported that pulling the reins out of the rider’s hands was the most frequent conflict behaviour in a group of show-jumping horses, but they also displayed head shaking, tail swishing, and mouth opening (gaping) [13]. These show-jumping horses tended to display a greater degree of problem behaviours associated with their heads and mouths, as the riders often shortened the reins abruptly. In the present study, conflict behaviours were generally shown more often in show-jumping horses than in dressage horses.

On the other hand, the relaxation behaviour playing with the bit (letting the bit move inside the mouth) only occurred significantly more with the 85°-ground-angle poll flexion position in show-jumping horses.

However, for both show-jumping and dressage horses, the most frequent relaxation behaviours with the 85°-ground-angle poll flexion position were ear play (ear movement) and having ears turned forward and not so much playing with the bit, even though the relaxation behaviours in general were shown more often by the show-jumping horses.

Overall, the discriminant analysis showed that by only using the results from the behaviour evaluation of this group of horses, it was possible to differentiate between the dressage and the show-jumping horses, as well as between the two poll flexion positions studied.

One possible explanation for the higher occurrence of conflict behaviours with the more flexed position in the show-jumping horses in the present study could be that they might not be so accustomed to being in this position for the same length of time as the dressage horses. It is interesting to observe that in the less flexed position (with only a 15^o^ difference), these same horses seemed to show a higher occurrence of relaxation behaviours. This is in agreement with the recommendations of the FEI to favour positioning the nose slightly in front of the vertical except for the moment at which the rider applies the aids, when the head may momentarily become more or less vertical. Although further studies need to be undertaken, we believe that the relevance of keeping the amount of time spent at a poll flexion position with a ground angle greater than 85° to an essential minimum should be further explored.

In the evaluation of the over-ground endoscopy videos, multiple **upper airway tract dynamic dysfunctions** were significantly more common when the 100°-ground-angle position was used in both equestrian disciplines. These were aryepiglottic fold axial deviation, palatal instability/dysfunction, and the collapse of various structures, such as the nasopharynx, vocal fold, intermittent bilateral arytenoid cartilage, and cricotracheal ligament. In show-jumping horses, ventroaxial luxation of the arytenoid corniculate process was also significantly more frequent with this degree of flexion.

Still, the highest scores for both the dressage and the show-jumping horses with the 100° ground angle were attributed to nasopharyngeal collapse, palatal instability/dysfunction, and intermittent bilateral arytenoid cartilage collapse.

The evaluation of upper respiratory tract dysfunctions as a cause of exercise intolerance has long been performed before specific recommendations are made for treatment in each equine athlete [27]. This phenomenon is referred to as dynamic obstruction of the upper respiratory tract in horses [28]. Not long ago, a comparative review was published reporting a similar situation identified more recently in human beings and classified as exercise-induced laryngeal obstruction (EILO) [29]. In humans with symptomatic EILO, females show higher resistance and higher degrees of laryngeal obstruction [30].

In horses, these dynamic abnormalities can only be seen during an exercising endoscopic evaluation, and the head and neck position may play a critical role in this process; therefore, the exact position during performance should be reproduced during the clinical examination to definitively define the abnormality [31]. This has been more easily achieved with the advent of over-ground endoscopy. Various authors state that there is strong evidence that head and neck position influences the occurrence of dorsal displacement of the soft palate and palatal instability [32,33,34,35], which is in agreement with the results obtained in the present study. Rakesh et al. (2008) showed that this was due to the most significant collapsing pressure being exerted on the floor of the rostral aspect of the nasopharynx during inhalation [36]. In addition, bilateral dynamic laryngeal collapse (the collapse of both arytenoid cartilages and vocal folds) during exercise is only manifested during exercising endoscopy with forced poll flexion [37,38]. Although it has been implied that clinical signs of dynamic laryngeal collapse associated with poll flexion could be induced when susceptible horses are ridden or driven into the bit, recent work could not find any clear evidence that the effect of a snaffle bit in a horse’s mouth, by breaking the airtight lip seal and by increasing pressure on the tongue, influenced the development or severity of the collapse, this being rather a consequence of the head and neck angles induced by rein tension [39]. A condition that has been appointed as a possible contribution to secondary nasopharyngeal collapse is alar fold collapse, as it causes mild to moderate expiratory obstruction [40]; however, this was not evaluated in the present study.

Although some upper airway abnormalities demonstrated an increase in occurrence in association with the more-flexed poll position, the effect of these upper airway abnormalities on the performance and welfare of dressage and show-jumping horses must be further evaluated.

When exercise intensity increases, there is a point at which the tissue demand for oxygen is greater than the respiratory and cardiovascular ability to supply it, leading to a significant component of anaerobic metabolism, with an increase in the levels of **lactate** tissue CO_2_. This is the lactate threshold [2]. In order to measure blood lactate concentration, an alternative approach to multiple blood collections is to collect after a single bout of submaximal exercise [41]. Previous authors found higher levels of blood lactate and higher HRs immediately after a trot and canter in horses ridden in hyperflexion than in the same horses ridden with light rein contact [17]. This increase in the flexed poll position also interferes with forward vision, which can contribute to anxiety and fear [5,10]. In addition to anxiety and fear, other psychological factors, such as excitement and pulling against the rider, may also result in elevated lactate and HR values [41,42,43].

In this study, the blood lactate levels were significantly higher after the dressage horses were ridden with the 100°-ground-angle poll flexion position (second test), but this was not true for the show-jumping horses. Still, when considering the blood samples from all horses together, the lactate levels were again significantly higher after they were ridden with the 100°-ground-angle poll flexion position. Apart from fatigue, another possible explanation for the significantly higher lactate values after the dressage horses’ second test is an association with psychological factors. In fact, ridder encouragement (whip, kicking, noise) and teeth grinding were only significantly more present when the 100°-ground-angle poll flexion position was used in this group of horses. There is a strong relationship between blood lactate and β endorphin levels as the latter is secreted to help tolerate the side effects of lactate accumulation through analgesia and enhancing happiness and motivation [44]. For horses, which are prey animals, stress-induced analgesia can be a component of prey–predator interactions that favours survival [45].

On the other hand, there was no significant difference in blood lactate levels between the first and the second tests in the show-jumping group. Some of our blood samples for lactate determination may have been collected too soon after exercise, as lactate levels may only peak from 5 min after exercise, which is the time lapse for its efflux from the muscle [42]. Otherwise, lactate can be considered an indicator of fatigue, so this group of show-jumping horses could have been more physically fit.

In show jumpers and dressage horses, field exercise testing is particularly valuable for the assessment of horses in which speed and the duration of exercise are not necessarily the most important factors to assess. For this submaximal exercise in sport horses, an indoor arena is preferred to reduce the likelihood of external influences affecting physiological measurements, such as **HR** [42], as we carried out in the present study.

In both equestrian disciplines studied, the HRs and **RRs** were significantly lower at the beginning of the second exercise test when compared to the beginning of the first, which could suggest that the horses became familiar with the space, the people, and the exercise test, as has been previously suggested [43]. Even so, they were higher at the end of the second exercise test when compared to the end of the first. Other authors have also reported changes in behavioural results and HR variability, which could be associated to exercise-induced stress due to artificial head–neck positions [12]. Nevertheless, HR variability has not yet been proven to be a measure of chronic pain [46].

**Pleural pressure (ΔPpl)** was significantly higher with the 100°-ground-angle position in both equestrian disciplines. This is in agreement with previous studies, which evaluated airway resistance with a balloon catheter in the oesophagus as well and found that it appeared to increase in all head and neck positions that were different from the natural head carriage and mostly in the hyperflexed position [18]. The significant reduction in the pharyngeal diameter found in the present study with the use of greater poll flexion in both equestrian disciplines likely contributed to these results.

**The pharyngeal diameter** was also significantly smaller at the 100° ground angle in both the dressage and show-jumping horses. Hyperflexion causes a significant reduction (5%) in the *rima glottidis* diameter [20]. The pharyngeal diameter in the dorsal flexed position was 29.6 +/− 11.3 mm in a previous study [16]. Some authors reported a diameter of 14.4–36.4 mm [47], and others described a diameter of 28.5 +/− 9.6 mm in the flexed position [21]. In the present study, the pharyngeal diameter at the 100° ground angle was even smaller, 11.62 +/− 5.0 mm for the dressage horses and 12.1 +/− 5.1 mm for the show jumpers in the 100° position, which could have been influenced by neck conformation. Depending on their anatomical conformation, different horses may find it more or less difficult to flex their neck and poll, so conformation is determinant for the severity of the impact of poll flexion on welfare. Still, additional research is necessary to assess the relevance of neck conformation for different equestrian disciplines [9,10,48]. Genetics, anatomy, and innervation seem to play a role in upper airway collapse, as there is some evidence that it can be related to breed, indicating an anatomic or functional cause, and it happens even in non-ridden horses [2,49]. In Norwegian–Swedish Coldblooded Trotters, genomic regions associated with dynamic laryngeal collapse have been identified [50]. Poll flexion produces conformational changes with respect to the relative positioning of the larynx and hyoid apparatus within the intermandibular space. The larynx is advanced more rostrally, decreasing airway lumen width [29,51].

Previous authors have contributed some suggestions. A modified check rein has been described to successfully limit poll flexion, preventing upper airway obstruction in Norwegian Coldblooded trotter racehorses [52,53]. Inspiratory muscle training has been shown to activate and induce a training response in the muscles of the upper airways and diaphragm in human athletes and has recently been explored in the horse [54]. Nevertheless, in a study evaluating the histopathology of intrinsic laryngeal musculature, the findings did not support a neuromuscular component within the pathogenesis of dynamic laryngeal collapse [55].

## 5. Conclusions

Based on the substantial number of studies previously conducted on the impact of hyperflexion on horse welfare, the ISES advised in a position paper for further research to be carried out on the physiological and psychological effects of lesser degrees of poll flexion and extension, which was the reason for the present study to be undertaken.

When testing this group of dressage and show-jumping horses with the 100°-ground-angle poll flexion positions, in comparison to the 85° poll flexion position, multiple upper airway dynamic dysfunctions were significantly more frequent, and the highest scores were attributed to nasopharyngeal collapse, palatal instability/dysfunction, and intermittent bilateral arytenoid cartilage collapse. In addition, most conflict behaviours were significantly more frequent at this poll flexion angle, with the highest scores attributed to excessive salivation (drooling), mouth opening, and turning the ears backward. On the other hand, it is also noteworthy that relaxation behaviours were significantly more frequent with the 85°-ground-angle poll flexion position, and the highest scores were attributed to turning the ears forward and ear play (ear movement). Although upper airway dysfunctions and conflict behaviours were more often associated with the more-flexed poll position, their effect on dressage and show-jumping horses’ performance and welfare must be further evaluated.

Furthermore, the pharyngeal diameter at the 100°-ground-angle poll flexion position was even smaller than the values previously reported in the literature, which probably contributed to the significantly higher pleural pressure (ΔPpl) also found with this more-flexed poll angle. This could have been influenced by neck conformation. Depending on their anatomical conformation, different horses may have more or less difficulty bending their neck and poll. Therefore, additional research is necessary to assess the relevance of neck conformation for different equestrian disciplines.

The most significant differences between the equestrian sports evaluated in the present study, dressage and show jumping, refer to the conflict and relaxation behaviours, as there were no significant differences in this respect for the upper airway dynamic dysfunctions. The fact that it was possible to discriminate between the two riding poll flexion positions based on a few conflict behaviours and a few relaxation behaviours could contribute to an easier detection of discomfort in the ridden horse.

Overall, the significant differences identified here for various parameters (the occurrence of upper airway dynamic dysfunctions and conflict behaviours, pharyngeal diameter, pleural pressure, and HR and RR) between two very close head and neck positions in the ridden horse support the idea that a variation of as little as 15° in poll flexion can have negative effects on the horses’ respiratory systems and behaviour and therefore on the horses’ welfare. We believe that the relevance of keeping the amount of time spent riding with a poll flexion ground angle greater than 85° to an essential minimum should be further explored for the benefit of horse performance and the horse’s quality of life in sport, thereby reassuring public concern.

## Figures and Tables

**Table 1:** Behaviour video classification, based on Smiet et al. (2014) and von Borstel et al. (2019). The 1st observation refers to the 1st riding test, with poll flexion at an 85° ground angle, and the 2nd observation refers to the 2nd riding test, with poll flexion at a 100° ground angle. Under the 1st and the 2nd observations, there are 3 separate spaces for each behaviour, which were intended to register the number of times the behaviours were shown during the 10-minute-long walk, the 15-minute-long trot, and the first 10 minutes of the gallop, respectively. These data were also registered as one final score for each behaviour: 0 (absent), 1 (occasional), 2 (moderately frequent or longer periods), 3 (considerably frequent or prolonged), or 4 (constant or continuous).

VIDEO BEHAVIOUR CLASSIFICATION Horse name ____________________ No. _____						
**Conflict Behaviour—Most Relevant**						
	1st Observation			2nd Observation		
Tail Swishing						
Head shaking						
Mouth opening						
Pulling the reins from the rider’s hands						
**Conflict Behaviour—other**						
	1st Observation			2nd Observation		
Ears—turned backwards						
-pinned back						
Head—lower (stretch forward + down)						
-raise						
-tilt						
-turn						
-pull in any direction when the reins are put in action						
Tongue—hang out						
-suck						
-lolly (in/out of mouth)						
Teeth—grind						
Mouth— tense (no bit movement, teeth occluded, tense jaw)						
-open or open/close (jaw movement)						
-excessive salivation/drooling						
Nostrils—open (more than neutral)						
-wide open						
Rearing/bucking/jumping						
**Relaxation Behaviour**						
	1st Observation			2nd Observation		
Ears—play (ear movement)						
-turned forward						
Bit—play (lets bit move inside mouth)						
-chew						
**Rider encouragement** (whip/kicking/sound)						
**Rider slowing down** (asks horse to move slower)						

**Table 2:** Means, SDs, and *p*-values of behaviours detected during exercise in the dressage horses, in the show-jumping horses, and in all horses together. First test with poll flexion at a ground angle of 85°. Second test with poll flexion at a ground angle of 100°.

Behaviour		Equestrian Discipline	First Test		Second Test		First vs. Second Tests
			Mean	SD	Mean	SD	*p*-Value
Conflict Behaviours— Most Relevant	Tail Swishing	Dressage	0.20	0.616	1.10	1.483	0.008
		Show Jumping	0.95	1.234	2.90	1.619	<0.001
		Show Jumping/Dressage (joint)	0.58	1.035	2.00	1.783	<0.001
		Show Jumping vs. Dressage	0.035		0.002		
	Head Shaking	Dressage	0.40	0.754	1.25	1.517	0.007
		Show Jumping	0.95	0.887	2.85	1.268	<0.001
		Show Jumping/Dressage (joint)	0.68	0.859	2.05	1.600	<0.001
		Show Jumping vs. Dressage	0.043		0.001		
	Mouth Opening	Dressage	1.15	0.988	2.95	0.759	<0.001
		Show Jumping	0.45	0.605	2.90	1.447	<0.001
		Show Jumping/Dressage (joint)	0.80	0.883	2.93	1.141	<0.001
		Show Jumping vs. Dressage	0.018		0.461		
Other Conflict Behaviours	Ears—turned backwards	Dressage	1.15	0.489	1.30	0.801	0.083
		Show Jumping	0.45	0.686	3.90	0.308	<0.001
		Show Jumping/Dressage (joint)	0.80	0.687	2.60	1.446	<0.001
		Show Jumping vs. Dressage	0.001		*p* < 0.001		
	Head—Raise	Dressage	0.10	0.447	0.15	0.671	0.102
		Show Jumping	0.30	0.571	1.55	1.605	0.005
		Show Jumping/Dressage (joint)	0.20	0.516	0.85	1.406	0.004
		Show Jumping vs. Dressage	0.314		0.002		
	Teeth Grinding	Dressage	0.10	0.308	1.30	1.867	0.014
		Show Jumping	0.00	0.000	0.00	0.000	1.00
		Show Jumping/Dressage (joint)	0.05	0.221	0.65	1.460	0.014
		Show Jumping vs. Dressage	0.602		0.600		
	Mouth—Open or Open/Close	Dressage	0.45	0.605	1.55	1.276	<0.001
		Show Jumping	0.45	0.999	2.50	1.701	<0.001
		Show Jumping/Dressage (joint)	0.45	0.815	2.03	1.561	<0.001
		Show Jumping vs. Dressage	0.445		0.076		
	Mouth—Excessive Salivation/ Drooling	Dressage	1.65	1.348	2.85	1.137	<0.001
		Show Jumping	0.85	1.268	2.85	1.599	<0.001
		Show Jumping/Dressage (joint)	1.25	1.354	2.85	1.369	<0.001
		Show Jumping vs. Dressage	0.068		0.602		
	Nostrils—Open (more than neutral)	Dressage	1.05	0.224	1.15	0.489	0.157
		Show Jumping	0.05	0.224	0.15	0.671	0.317
		Show Jumping/Dressage (joint)	0.55	0.224	0.65	0.585	0.098
		Show Jumping vs. Dressage	*p* < 0.001		*p* < 0.001		
	Rearing/Bucking/Jumping	Dressage	0.10	0.308	0.25	0.444	0.180
		Show Jumping	0.20	0.410	0.55	0.686	0.035
		Show Jumping/Dressage (joint)	0.15	0.362	0.40	0.591	0.012
		Show Jumping vs. Dressage	0.602		0.231		
Relaxation Behaviour	Ears—Play (ear movement)	Dressage	1.00	0.000	0.15	0.366	<0.001
		Show Jumping	3.90	0.308	0.25	0.550	<0.001
		Show Jumping/Dressage (joint)	2.45	1.484	0.20	0.464	<0.001
		Show Jumping vs. Dressage	*p* < 0.001		0.758		
	Ears—Turned forward	Dressage	1.00	0.000	0.70	0.470	0.014
		Show Jumping	4.00	0.000	0.70	0.571	<0.001
		Show Jumping/Dressage (joint)	2.50	1.519	0.70	0.516	<0.001
		Show Jumping vs. Dressage	*p* < 0.001		0.947		
	Bit—Play (letting the bit move inside the mouth)	Dressage	0.00	0.000	0.00	0.000	1.000
		Show Jumping	1.55	1.638	0.15	0.489	0.004
		Show Jumping/Dressage (joint)	0.78	1.387	0.08	0.350	0.004
		Show Jumping vs. Dressage	0.006		0.602		
Rider Encouragement (whip/ kicking/noise)		Dressage	0.30	0.571	0.65	0.813	0.034
		Show Jumping	0.45	0.686	0.95	1.050	0.065
		Show Jumping/Dressage (joint)	0.38	0.628	0.80	0.939	0.007
		Show Jumping vs. Dressage	0.565		0.445		

**Table 3:** Means, SDs, and *p*-values of upper airway tract dysfunctions detected via over-ground endoscopy during exercise in the dressage horses, in the show-jumping horses, and in all horses together. Show jumping vs. dressage is not shown as no significant differences were found for any of the parameters. First test with poll flexion at a ground angle of 85°. Second test with poll flexion at a ground angle of 100°.

Upper Airway Dysfunctions	Equestrian Discipline	First Test		Second Test		First vs. Second Test
		Mean	SD	Mean	SD	*p*-Value
Aryepiglottic Folds (axial deviation)	Dressage	0.35	0.489	0.75	0.639	0.011
	Show Jumping	0.55	0.605	0.70	0.733	0.03
	Show Jumping/Dressage (Joint)	0.45	0.552	0.73	0.679	0.02
Corniculate Process of Arytenoid (ventro-axial luxation)	Dressage	0.65	0.813	0.80	0.894	0.180
	Show Jumping	0.55	0.759	0.85	0.671	0.014
	Show Jumping/Dressage (Joint)	0.60	0.778	0.83	0.781	0.007
Palatal Instability/ Dysfunction (PI)	Dressage	1.05	0.759	1.60	0.681	0.005
	Show Jumping	0.85	0.587	2.05	0.394	0.000
	Show Jumping/Dressage (Joint)	0.95	0.677	1.83	0.594	<0.001
Intermittent Bilateral Arytenoid Cartilage Collapse	Dressage	0.85	0.933	1.95	1.234	0.000
	Show Jumping	0.90	1.021	1.30	1.302	0.038
	Show Jumping/Dressage (Joint)	0.88	0.966	1.63	1.295	<0.001
Vocal Fold Collapse	Dressage	0.60	0.598	0.95	0.605	0.001
	Show Jumping	0.80	0.616	1.10	0.718	0.034
	Show Jumping/Dressage (Joint)	0.70	0.608	1.03	0.660	0.01
Nasopharyngeal Collapse	Dressage	0.55	0.887	1.95	1.605	0.002
	Show Jumping	0.60	1.142	2.50	1.606	0.001
	Show Jumping/Dressage (Joint)	0.58	1.010	2.23	1.609	<0.001
Cricotracheal ligament collapse	Dressage	0.05	0.224	0.25	0.444	0.046
	Show Jumping	0.15	0.366	0.35	0.745	0.046
	Show Jumping/Dressage (Joint)	0.10	0.304	0.30	0.608	0.005
Pharyngeal Lymphoid Hyperplasia	Dressage	0.70	0.657	0.90	0.912	0.046
	Show Jumping	0.65	0.813	0.70	0.801	0.317
	Show Jumping/Dressage (Joint)	0.68	0.730	0.80	0.853	0.025

**Table 4:** Mean heart and respiratory rates for dressage horses, for show-jumping horses, and for all horses together in the first and second tests, before and after each test. In the rows, different letters correspond to significantly different means (*p* < 0.05).

Heart Rate	First Test				Second Test				
	Before		After		Before		After		First vs. Second Test
	Mean	SD	Mean	SD	Mean	SD	Mean	SD	
Dressage	46.90 a	6.398	93.95 b	11.325	41.50 a	6.083	108.25 c	14.671	*p* < 0.001
Show Jumping	51.90 b	4.887	85.50 c	12.292	44.35 a	4.998	99.00 d	11.814	*p* < 0.001
All Horses Together	49.40	6.164	89.73	12.426	42.93	5.681	103.63	13.957	*p* < 0.001
Dressage vs. Show Jumping (*p*)	0.02		0.01		0.265		0.04		
Respiratory Rate	First test				Second test				
	Before		After		Before		After		First vs. second test
	Mean	SD	Mean	SD	Mean	SD	Mean	SD	
Dressage	23.40 a	7.170	65.35 b	18.199	20.30 a	6.027	79.40 c	14.637	*p* < 0.001
Show Jumping	34.00 b	12.329	64.95 c	24.412	27.45 a	10.081	82.60 d	22.281	*p* < 0.001
All Horses Together	28.70	11.310	65.15	21.254	23.88	8.962	81.00	18.678	*p* < 0.001
Dressage vs. Show Jumping (*p*)	0.003		0.904		0.009		0.383		

## Data Availability

The data presented in this study are available upon request from the corresponding author. The data are not publicly available due to a privacy request from the horses’ legal owners.
